# Referral pathways as mechanisms of child maltreatment identification: evidence from a German Childhood-House

**DOI:** 10.3389/frcha.2026.1907809

**Published:** 2026-08-28

**Authors:** Silke Pawils, Elisa Grossmann, Axel Heinemann, Benjamin Ondruschka

**Affiliations:** 1 Institute and Polyclinic for Medical Psychology, University Medical Center Hamburg-Eppendorf, Hamburg, Germany; 2 Department of General Pediatrics, Neonatology and Pediatric Cardiology, Medical Faculty and University Hospital Düsseldorf, Heinrich Heine University Düsseldorf, Düsseldorf, Germany; 3 Department of Cardiology, University Medical Center Hamburg-Eppendorf, University Heart & Vascular Center Hamburg, Hamburg, Germany; 4 Center for Population Health Innovation (POINT), University Heart & Vascular Center Hamburg, University Medical Center Hamburg-Eppendorf, Hamburg, Germany; 5 Institute of Molecular Biology (IMB), Mainz, Germany; 6 Institute of Legal Medicine, University Medical Center Hamburg-Eppendorf, Hamburg, Germany

**Keywords:** ACE-related child maltreatment profiles, Child Advocacy Center, child maltreatment, child protection, Childhood-House, interdisciplinary collaboration, referral pathways

## Abstract

**Background:**

Adverse childhood experiences-related child maltreatment, including abuse and neglect, are major threats to children's safety, health, and development. Their identification depends on recognition and referral processes occurring across multiple professional systems. Childhood-Houses, based on Child Advocacy Center and Barnahus principles, provide multidisciplinary assessment and support for children with suspected maltreatment experiences.

**Objective:**

This study examined whether identified child maltreatment profiles and multidisciplinary assessment processes differed according to referral pathways to a German Childhood-House. In addition, perspectives of external referrers regarding interdisciplinary collaboration were explored.

**Methods:**

A quantitative observational study was conducted using two complementary data sources. Routine documentation data from children and adolescents referred to the Childhood-House Hamburg between January and September 2023 were analyzed (*n* = 731). Furthermore, an exploratory online survey among external referrers from youth welfare services, healthcare, and law enforcement agencies was conducted in the same period (*n* = 66). Descriptive and exploratory analyses examined differences in referral pathways, identified maltreatment profiles, multidisciplinary assessment procedures, and collaboration experiences.

**Results:**

Referral pathways were associated with distinct child maltreatment profiles. Medical, physical, and emotional neglect were identified predominantly among youth welfare referrals, while physical abuse and sexualized violence occurred more frequently among healthcare and law enforcement referrals. Multidisciplinary assessment procedures also differed across referral pathways. Referrers reported generally positive collaboration experiences but identified needs for improvement regarding psychological assessment, feedback procedures, and interdisciplinary communication.

**Conclusions:**

The findings indicate that referral pathways serve as important mechanisms of child maltreatment identification and influence access to multidisciplinary child protection services. Strengthening standardized identification procedures, referral structures, and cross-sector collaboration may support more comprehensive responses to ACE-related child maltreatment experiences.

## 1 Introduction

Child maltreatment, including physical abuse, emotional abuse, sexualized violence, and neglect, represents a major public health and child protection concern. These adverse childhood experiences (ACE)-related maltreatment experiences are associated with adverse developmental, mental, physical, and social outcomes and therefore require timely identification and intervention (1, 2). Population-based studies indicate that child maltreatment is common and frequently co-occurring. In contrast, official child protection statistics likely underestimate its true prevalence because identification depends on recognition, reporting, and referral processes across different professional systems (3–5).

The identification of child maltreatment occurs within complex child protection systems involving healthcare providers, youth welfare services, law enforcement agencies, educational institutions, and families. Professional groups differ in their responsibilities, opportunities for observation, and reporting practices. Consequently, referral pathways may influence which forms of maltreatment become visible and subsequently receive specialized assessment (3, 6, 7). Previous research suggests that healthcare and law enforcement agencies more frequently identify physical abuse and sexualized violence, while youth welfare services are more likely to encounter neglect and chronic family adversity (3, 5). However, empirical evidence on differences in identified maltreatment profiles across referral pathways remains limited.

To address the complexity of child maltreatment, multidisciplinary Child Advocacy Center models have been established internationally. In Germany, Childhood-Houses are based on Child Advocacy Center and Barnahus principles and provide coordinated pediatric, forensic, psychological, and social assessments for children with suspected maltreatment experiences (8, 9). Because Childhood-Houses receive referrals from multiple professional sectors, they provide an opportunity to examine how referral pathways contribute to the identification of child maltreatment and shape subsequent assessment and care processes.

Despite the increasing implementation of Childhood-Houses and other multidisciplinary child protection models, little is known about whether children referred through different pathways present distinct maltreatment profiles and whether multidisciplinary assessment procedures differ accordingly. Furthermore, evidence regarding referrers' experiences of interdisciplinary collaboration remains scarce.

A fundamental prerequisite for the identification of child maltreatment is the existence of structured and standardized data collection systems. Reliable documentation of suspected and confirmed maltreatment cases enables the systematic recognition of patterns, facilitates communication across pro-fessional sectors, and provides the empirical basis for monitoring child protection needs. In many countries, however, child maltreatment data are collected across fragmented healthcare, welfare, educational, and law enforcement systems using different documentation standards. As a result, substantial numbers of affected children may remain invisible within official statistics, and opportunities for early identification can be missed. Multidisciplinary child protection ser-vices such as Childhood-Houses are uniquely positioned to contribute to more standardized (and reproducible) documentation practices because they integrate information from multiple referral sources and professional perspectives. Understanding how children enter these systems is therefore not only relevant for service provision but also for improving the quality and completeness of child maltreatment surveillance and data collection.

Using these routine documentation data from the Childhood-House Hamburg and survey data from external referrers, this study pursued three objectives: (i) to examine whether identified child maltreatment profiles differ across referral pathways; (ii) to investigate whether multidisciplinary assessment processes vary according to referral pathway; and (iii) to explore how referring professionals evaluate interdisciplinary collaboration and communication. Referral pathways were conceptualized as mechanisms through which ACE-related child maltreatment experiences become visible within child protection systems.

## 2 Methods

### 2.1 Study design and setting

This observational study combined routine documentation data from the Childhood-House Hamburg with survey data from external referring professionals. The Childhood-House Hamburg is a multidisciplinary child protection competency center based on Child Advocacy Center and Barnahus principles. It provides coordinated assessments for children and adolescents with suspected maltreatment experiences through collaboration between pediatric, forensic, psychological, and social service professionals.

The study examined whether identified child maltreatment profiles and multidisciplinary assessment procedures differed according to referral pathway. In addition, the study explored referring professionals' experiences of collaboration with the Childhood-House.

### 2.2 Participants and data sources

#### 2.2.1 Routine documentation data

Routine documentation data from all children and adolescents referred to Childhood-House Hamburg between January and September 2023 were included in the analyses. During the study period, 731 children and adolescents were referred to the institution; with 531 completed diagnostic assessments.

For each case, information on demographic characteristics, referral source, identified maltreatment categories, and multidisciplinary assessment procedures was extracted from the electronic documentation system. Referral pathways were categorized as youth welfare services, healthcare services, law enforcement agencies, or private referrals. The analyses of maltreatment profiles were conducted on the 531 cases with complete diagnostic assessments.

#### 2.2.2 Referrer survey

To complement the documentation data, an online survey was conducted among professionals who had referred at least one child to the institution. Eligible participants were contacted via email and invited to complete an anonymous questionnaire.

The survey assessed professional background characteristics, referral experiences, collaboration with Childhood-House Hamburg, communication processes, satisfaction with services, and perceived opportunities for improvement. Both closed-ended and open-ended questions were included. A total of 66 professionals participated in the survey.

### 2.3 Measures

#### 2.3.1 Child maltreatment categories

Child maltreatment categories were based on multidisciplinary assessments documented during routine practice. The categories included physical abuse, emotional abuse, sexualized violence, physical neglect, emotional neglect, and medical neglect. Multiple categories could be assigned to the same child if more than one form of maltreatment was identified.

For the purposes of this study, all these categories were considered ACE-related child maltreatment experiences and were analyzed separately as well as in combination.

#### 2.3.2 Multidisciplinary assessment procedures

Information on assessment and support procedures was extracted from routine documentation. These procedures included initial pediatric or forensic examinations, follow-up examinations, professional counseling, expert reports, psychological assessments, and child protection conferences.

Because assessment procedures are tailored to individual case characteristics, not all children received the same interventions. The present analyses therefore focused on whether specific procedures were documented during the assessment process.

#### 2.3.3 Referrer experiences

The survey further examined experiences of interdisciplinary collaboration. Participants evaluated communication, accessibility, referral procedures, and overall collaboration with Childhood-House Hamburg. Additional open-ended questions allowed respondents to describe strengths of the service and identify areas for further development in a more personal opinion.

### 2.4 Statistical analysis

All analyses were conducted using R-Studio (Posit PBC, Boston, US). Descriptive statistics were calculated for demographic characteristics, referral pathways, maltreatment categories, multidisciplinary assessment procedures, and survey responses.

Differences between referral pathways were examined using Chi-square or Fisher's exact tests for variables on a nominal scale and Kruskal–Wallis tests for ordinal variables. Dunn-posthoc tests with Bonferroni correction were carried out to address pairwise differences. In addition, Cramer's V and Cohen's r were calculated as effect measures depending on variable scaling. Effect sizes were reported alongside significance tests, however *p*-values were understood rather descriptively due to the exploratory nature of the study and multiple testing.

## 3 Results

### 3.1 Sample characteristics

#### 3.1.1 Characteristics of children referred to Childhood-House Hamburg

A total of 731 children and adolescents referred to Childhood-House Hamburg between January and September 2023 were included in the analyses. The largest referral group originated from youth welfare offices (64.2%; *n* = 469), followed by private referrals (18.5%; *n* = 135), healthcare services (10.3%; *n* = 75), and law enforcement agencies (7.1%; *n* = 52).

Overall, 51.7% (*n* = 374) of referred children were female. The proportion of female children differed across referral pathways and ranged from 46.3% among youth welfare referrals to 69.3% among healthcare referrals.

Age distributions also varied between referral pathways. Children aged 1–4 years and 5–9 years represented the largest age groups overall. Adolescents aged 15–17 years accounted for a larger proportion of law enforcement and private referrals than of youth welfare referrals (see Table 1).

**Table 1:** Demographic characteristics of children and adolescents with suspected child maltreatment experiences by access pathway to the Childhood-House Hamburg: *n* (%) and number of missings (n_miss_).

Sample characteristics	*n* (%)				
	Total	YWO	Healthcare	LEA	Private/others
Sex*					
Female	374 (51.7%)	226 (48.2%)	52 (69.3%)	31 (59.6%)	65 (50.8%)
Male	350 (48.3%)	243 (51.8%)	23 (30.7%)	21 (40.4%)	63 (49.2%)
*n*_miss_	7	0	0	0	7
Age					
<1 year	51 (7.3%)	23 (4.9%)	16 (22.5%)	1 (2.0%)	11 (9.7%)
1–4 years	198 (28.2%)	145 (31.1%)	21 (29.6%)	8 (15.7%)	24 (21.2%)
5–9 years	218 (31.1%)	176 (37.8%)	10 (14.1%)	10 (19.6%)	22 (19.5%)
10–14 years	127 (18.1%)	81 (17.5%)	8 (11.3%)	13 (25.5%)	25 (22.1%)
15–17 years	104 (14.8%)	40 (8.6%)	16 (22.5%)	19 (37.3%)	29 (25.7%)
18 years and older	3 (0.4%)	1 (0.2%)	0 (0.0%)	0 (0.0%)	2 (1.8%)
*n*_miss_	30	3	4	1	22
Total *n* (%)	731 (100.0%)	469 (64.2%)	75 (10.3%)	52 (7.1%)	135 (18.5%)

YWO, youth welfare offices; LEA, law enforcement agencies.

* No diverse identity.

#### 3.1.2 Characteristics of referring professionals

Sixty-six professionals participated in the referrer survey. Most respondents worked in youth welfare services (78.8%), followed by healthcare services (10.6%) and law enforcement agencies (10.6%). The mean age of respondents was 38.80 years (SD = 8.63), and the mean duration of professional experience was 8.2 years (SD = 6.6).

Most respondents reported previous child protection training and referral experiences (see Table 2).

**Table 2:** Demographic characteristics of referrers to the Childhood-House Hamburg by referral pathway: *n* (%), M (SD) and number of missings (n_miss_).

Sample characteristics	n (%)/M (SD)			
	Total	YWO	Healthcare	LEA
Sex of referrers*				
Female	54 (87.1%)	47 (94.0%)	4 (66.7%)	3 (50.0%)
Male	8 (12.9%)	3 (6.0%)	2 (33.3%)	3 (50.0%)
*n*_miss_	4	2	1	1
Age of referrers in years	38.80 (8.63)	38.22 (8.71)	44.00 (5.06)	38.33 (10.13)
*n*_miss_	5	3	1	1
Professional experience of referrers in years	8.24 (6.63)	7.24 (5.65)	11.83 (4.83)	13.00 (12.17)
*n*_miss_	4	2	1	1
Training in child protection				
Yes	60 (90.9%)	51 (98.1%)	7 (100.0%)	2 (28.6%)
No	6 (9.1%)	1 (1.9%)	0 (0.0%)	5 (71.4%)
*n*_miss_	0	0	0	0
Total *n* (%)	66 (100.0%)	52 (78.8%)	7 (10.6%)	7 (10.6%)

YWO, youth welfare offices; LEA, law enforcement agencies.

* No diverse identity.

### 3.2 Identified child maltreatment profiles across referral pathways

For a total of 531 children, multidisciplinary maltreatment assessment was conducted and documented. Maltreatment was confirmed in 37.7% of cases. The proportion of confirmed maltreatment differed across referral pathways and was highest among youth welfare referrals (46.3%), followed by healthcare (24.0%), law enforcement (19.1%), and private referrals (15.5%).

Significant differences were observed in the distribution of identified maltreatment categories. Neglect-related maltreatment, including medical, physical, and emotional neglect, was documented predominantly among children referred by youth welfare services. Physical neglect was identified exclusively within this referral pathway. In contrast, physical abuse occurred most frequently among healthcare referrals, whereas confirmed sexualized violence was documented only among law enforcement referrals. Cases involving violence perpetrated by third parties were observed primarily among healthcare and law enforcement referrals.

The proportion of children with multiple confirmed maltreatment categories also differed across referral pathways and was highest among youth welfare referrals. Detailed frequencies and statistical comparisons are presented in Table 3.

**Table 3:** Exploratory tests on differences of diagnostic outcomes by access pathway, Chi-squared and fisher's exact tests with test values: *χ*^2^ (degrees of freedom) with *p*-values and pairwise posthoc tests with Bonferroni correction and cramer's V.

Exploratory tests: Test statistics	Cramer's *V* (*p*-value)					
	YWO vs. Healthcare	YWO vs. LEA	YWO vs. Private	Healthcare vs. LEA	Healthcare vs. Private	LEA vs. Private
Child maltreatment (total) *χ*^2^ (6) = 78.84 (*p* < 0.001)^a^	0.22 (*p* < 0.001)	0.24 (*p* < 0.001)	0.37 (*p* < 0.001)	0.06 (*p* = 0.999)	0.18 (*p* = 0.340)	0.16 (*p* = 0.999)
Physical violence *χ*^2^ (9) = 152.47 (*p* < 0.001)^b^	0.22 (*p* < 0.001)	0.49 (*p* < 0.001)	0.58 (*p* < 0.001)	0.39 (*p* = 0.033)	0.44 (*p* = 0.002)	0.21 (*p* = 0.999)
Emotional abuse *χ*^2^ (9) = 53.30 (*p* < 0.001)^b^	0.07 (*p* = 0.999)	0.15 (*p* = 0.822)	0.48 (*p* < 0.001)	0.39 (*p* = 0.999)	0.59 (*p* = 0.102)	0.43 (*p* = 0.768)
Sexualized violence *χ*^2^ (9) = 27.79 (*p* < 0.001)^b^	-/- ^c^	0.65 (*p* < 0.001)	-/- ^c^	0.45 (*p* = 0.588)	-/- ^c^	0.60 (*p* = 0.148)
Medical neglect *χ*^2^ (6) = 39.59 (*p* < 0.001)^b^	0.21 (*p* = 0.002)	0.18 (*p* = 0.007)	0.22 (*p* < 0.001)	0.15 (*p* = 0.999)	-/- ^c^	0.16 (*p* = 0.999)
Physical neglect *χ*^2^ (6) = 15.24 (*p* = 0.010)^b^	0.12 (*p* = 0.322)	0.10 (*p* = 0.738)	0.14 (*p* = 0.150)	-/- ^c^	-/- ^c^	-/- ^c^
Emotional neglect *χ*^2^ (6) = 11.48 (*p* = 0.060)^b^	0.09 (*p* = 0.999)	0.09 (*p* = 0.999)	0.20 (*p* = 0.049)	-/- ^c^	0.29 (*p* = 0.999)	0.50 (*p* = 0.103)
Number of diagnoses *χ*^2^ (6) = 39.61 (*p* < 0.001)^a^	0.09 (*p* = 0.999)	0.09 (*p* = 0.999)	0.20 (*p* = 0.077)	-/- ^c^	0.29 (*p* = 0.999)	0.50 (*p* = 0.106)

YWO, youth welfare offices; LEA, law enforcement agencies.

*p* < 0.05 was considered statistically significant.

a Chi-Squared tests.

b Fisher's exact tests.

c No pairwise test possible due to absence of cases in both comparison groups.

### 3.3 Multidisciplinary assessment and support processes across referral pathways

Multidisciplinary assessment procedures differed across referral pathways. An initial pediatric or forensic assessment was documented for 70.9% of all children and occurred most frequently among law enforcement referrals (88.5%) and youth welfare referrals (76.1%). Follow-up examinations were documented primarily among youth welfare referrals (24.5%).

**Figure 1:**
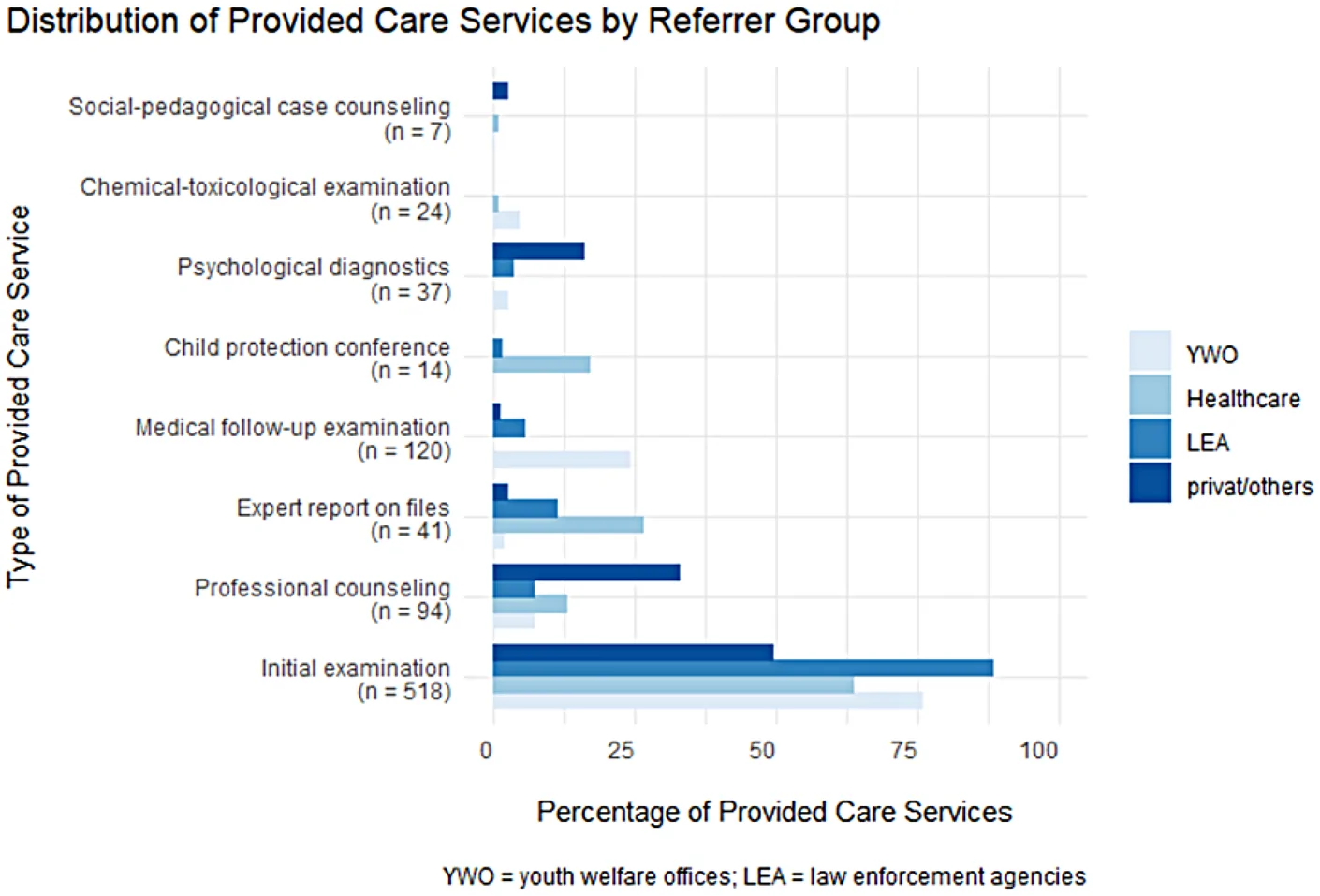
Differences in the distribution of provided care service of suspected child maltreatment cases by access pathway to the Childhood-House Hamburg.

Professional counseling was most common among privately referred children (33.3%), whereas expert reports were documented most frequently among healthcare referrals (26.7%). Psychological assessments were comparatively rare overall (5.1%) and occurred most often among private referrals (16.3%). Child protection conferences were documented infrequently (1.9%) but were most common among healthcare referrals (17.3%) (see Figure 1).

### 3.4 Referrer experiences with collaboration and communication

Overall, referrers reported good experiences with the Childhood-House Hamburg. Across professional sectors, ratings of referral procedures, personal communication, and collaboration were predominantly located in the positive range of the response scales.

**Figure 2:**
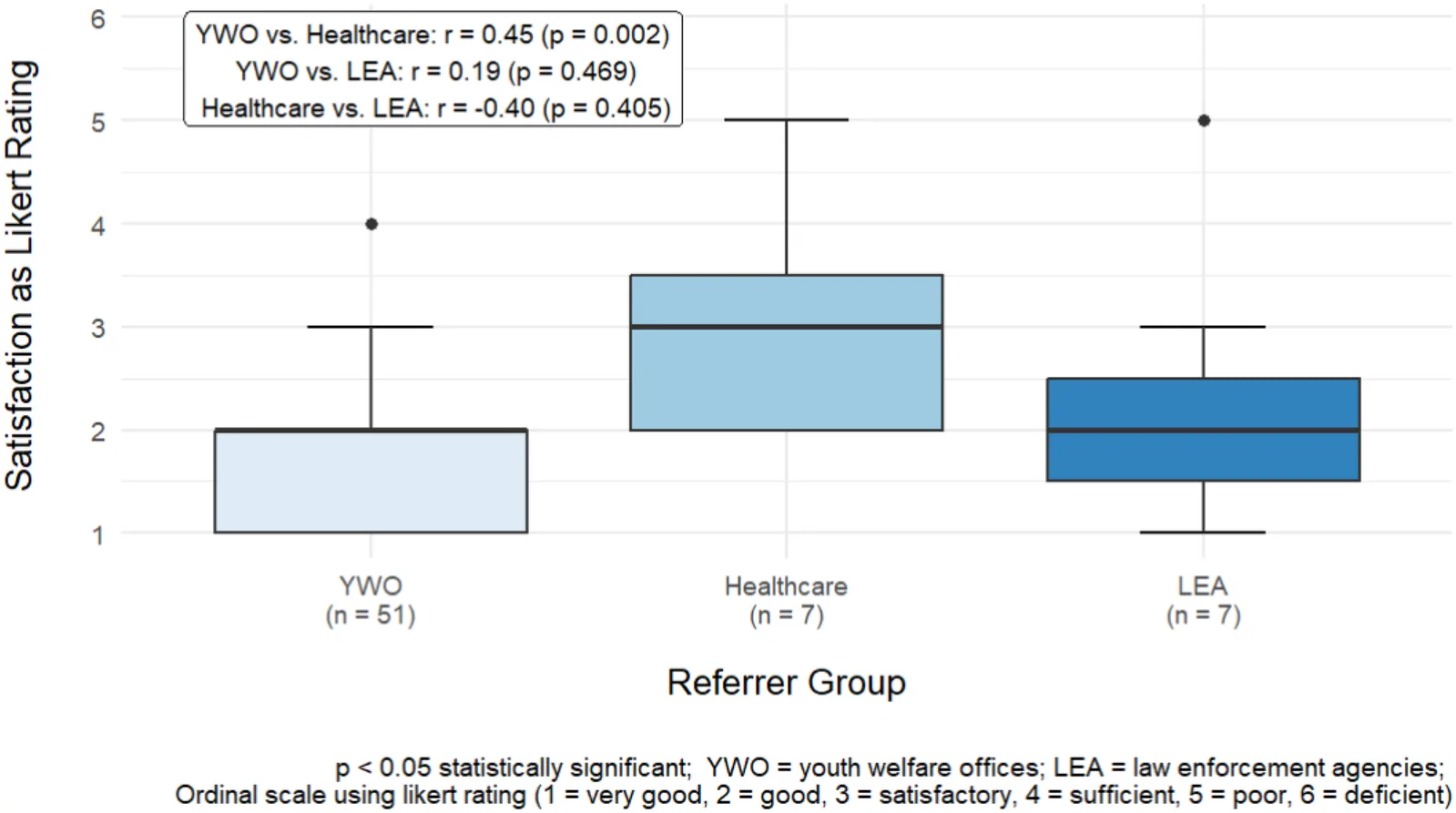
Differences in the distribution of satisfaction with referral process of suspected child maltreatment cases by referral pathway to the Childhood-House Hamburg with pairwise posthoc tests: effect size r and *p*-value with Bonferroni correction.

Willingness to refer children to the Childhood-House in the future and to recommend the service to colleagues was high across all referral groups. At the same time, variation between professional sectors was observed for selected aspects of collaboration. In particular, lower ratings were reported regarding feedback after referrals and information on case outcomes, especially among respondents from healthcare services and law enforcement agencies (see Figure 2).

Differences across referral pathways were observed regarding identified maltreatment profiles, multidisciplinary assessment procedures, and collaboration experiences. The following discussion considers these findings in relation to existing research on child maltreatment identification**,** referral mechanisms, and multidisciplinary child protection systems.

## 4 Discussion

### 4.1 Referral pathways as mechanisms of child maltreatment identification

The present study examined whether identified child maltreatment profiles and multidisciplinary assessment procedures differed according to referral pathways to a German Childhood-House. Across all analyses, referral source was associated with substantial differences in both identified maltreatment categories and assessment processes. Rather than representing neutral administrative routes into services, referral pathways appeared to be closely linked to how concerns became recognized and subsequently assessed within the child protection system.

A central observation was that children referred from different sectors did not present with the same patterns of identified maltreatment. Youth welfare referrals were characterized by higher proportions of neglect-related maltreatment and multiple maltreatment categories, whereas healthcare and law enforcement referrals were more strongly associated with physical abuse and sexualized violence. Similar patterns have been described in previous studies indicating that different professional systems identify different segments of the broader population of maltreated children (3, 5).

Importantly, these findings should not be interpreted as evidence that specific forms of maltreatment occur predominantly within one service context. Rather, the findings suggest that different professional systems provide different opportunities for recognition. Child maltreatment becomes visible only when concerns are observed, interpreted, and acted upon by adults or caregivers who have access to children and families. Consequently, identification depends not only on the experiences of children themselves but also on the institutional settings in which concerns emerge.

One possible explanation for the observed differences lies in the distinct roles of the professional sectors involved in child protection. Youth welfare services often maintain longer-term contact with children and families and may therefore be more likely to observe chronic patterns of adversity. Healthcare professionals, in contrast, frequently encounter children in situations involving injuries, acute health concerns, or developmental problems. Law enforcement agencies typically become involved when criminal investigations or formal disclosures have already occurred. These different opportunities for observation may influence which concerns are recognized and subsequently referred for specialized assessment.

From a systems perspective, the findings support the view that identification is not a single event but a process distributed across multiple institutions. Referral pathways may therefore be understood as part of the identification process itself. The route through which a child enters a multidisciplinary service appears to influence which forms of maltreatment are documented, which assessments are conducted, and which professional perspectives become involved.

This interpretation is particularly relevant for multidisciplinary child protection models such as Childhood-Houses. Because these services receive referrals from multiple sectors, they provide opportunities to integrate perspectives that would otherwise remain separated across institutional boundaries. The present findings therefore suggest that the value of multidisciplinary child protection services extends beyond coordinated assessment alone. They may also act as points of convergence within fragmented child protection systems.

### 4.2 Different professional systems detect different forms of maltreatment

One of the most striking findings concerned the concentration of neglect-related maltreatment among referrals from youth welfare services. Medical neglect, physical neglect, and emotional neglect were identified predominantly among children referred through this pathway, whereas such cases were considerably less common among healthcare, law enforcement, or private referrals.

This pattern is consistent with previous research suggesting that neglect differs from other forms of maltreatment in both presentation and detection (3, 5, 8, 9). Unlike physical abuse or sexualized violence, neglect often develops gradually and may not be associated with a single identifiable incident. Instead, concerns frequently emerge through the accumulation of observations regarding supervision, healthcare utilization, developmental support, or emotional availability. Professionals who maintain continuous contact with families may therefore be particularly likely to identify such patterns and are more prone to this type of abuse than institutions with only single contacts to the familiar setting.

The concentration of neglect among youth welfare referrals may reflect these characteristics. Youth welfare professionals often work with families over extended periods and may observe recurring indicators that would be difficult to recognize during isolated encounters. The findings therefore support the assumption that different forms of maltreatment require different opportunities for observation and assessment.

A different pattern emerged for physical abuse. Cases of physical abuse were identified most frequently among healthcare referrals. This finding corresponds with previous evidence highlighting the important role of healthcare providers in recognizing injuries that may be indicative of maltreatment (6, 10, 11). Physical injuries often create an immediate reason for professional assessment including forensic interpretation of the type of injury and may therefore increase opportunities for detection. Healthcare settings can consequently function as important gateways into child protection systems for children affected by physical violence.

Sexualized violence showed yet another pattern and was documented primarily among law enforcement referrals. Although the present study was not designed to examine disclosure processes directly, this finding may reflect the close connection between disclosures of sexualized violence and formal investigative procedures. In many cases, police involvement occurs shortly after disclosure and may therefore represent another important pathway into multidisciplinary assessment services. Similar observations have been reported in previous research examining referral structures within child protection systems (3, 5).

Taken together, the findings indicate that professional systems differ not only in the number of children referred but also in the forms of maltreatment that become visible within their respective contexts. This observation has important implications, as reliance on a single referral source may result in systematic under-identification of specific maltreatment experiences. Comprehensive child protection therefore requires collaboration across systems that contribute complementary opportunities for recognition and referral.

### 4.3 Strengths and limitations

Several limitations should be considered when interpreting the findings. Most importantly, the study was conducted within a single yet large Childhood-House in Germany and therefore reflects one specific organizational and regional context. Referral structures, service availability, and professional responsibilities may differ across jurisdictions, limiting the generalizability of the results.

A second consideration concerns the use of routine documentation data. Although routine data provide valuable insights into real-world child protection practice, they are not collected primarily for research purposes. Variations in documentation procedures and assessment practices cannot be excluded. Similarly, the exploratory nature of the analyses means that findings should be interpreted as hypothesis-generating rather than definitive evidence regarding causal mechanisms.

The survey component is also subject to limitations. Participation was voluntary, and it is possible that professionals with particularly positive or negative experiences were more likely to respond. Furthermore, some referral groups were comparatively small, especially within healthcare and law enforcement sectors, which limits the precision of subgroup comparisons.

Another important consideration concerns the scope of the study. The analyses focused on identification and assessment processes rather than developmental outcomes, psychopathology, or intervention effectiveness. Consequently, the findings provide insight into how children gain access to multidisciplinary services but do not permit conclusions regarding longer-term outcomes following assessment or adequate support.

Although referral pathways were associated with distinct maltreatment profiles, differences in age and sex distributions across referral groups may have contributed to some of the observed patterns. As no multivariable analyses adjusting for these characteristics were conducted, the findings should be interpreted with caution. Future studies should examine whether the observed associations remain after adjustment for demographic factors.

Despite these limitations, the study has several notable strengths. To our knowledge, it is among the first investigations to examine referral pathways, identified maltreatment profiles, multidisciplinary assessment procedures, and referrer perspectives within a German Childhood-House. Furthermore, the combination of routine documentation data and survey data allowed the integration of institutional and professional perspectives. This mixed-source approach provides a more comprehensive understanding of child protection processes than either data source alone.

Perhaps most importantly, the study shifts attention toward an aspect of child protection that has received comparatively limited empirical attention to date. While much of the literature focuses on prevalence, risk factors, or consequences of maltreatment, the present study highlights the mechanisms through which children become identified and referred for specialized support in competence centers. Understanding these processes is a key prerequisite for improving access to protection and care.

### 4.4 Implications for multidisciplinary child protection practice

The findings have several implications for practice. First, they suggest that no single professional sector can be expected to identify all forms of child maltreatment equally well. Different systems appear to contribute distinct perspectives that reflect their professional responsibilities and opportunities for observation. Efforts to improve child protection may therefore benefit from strengthening coordination across sectors rather than focusing exclusively on individual professions.

Second, the results underline the importance of multidisciplinary assessment models. Because different referral sources were associated with different maltreatment profiles, comprehensive assessments should extend beyond the immediate reason for referral whenever possible. Children referred for one concern may also have experienced additional forms of maltreatment that would otherwise remain unrecognized.

Third, the survey findings point to the continuing importance of communication and feedback within multidisciplinary networks. Although overall satisfaction with Childhood-House Hamburg was high, respondents identified opportunities for improvement regarding information exchange and follow-up communication indicating the necessity to define and respect a “common language” between specialists' group. Strengthening these processes may contribute to more effective collaboration between referring institutions and specialized child protection services.

The findings also highlight the importance of structured data collection points within child protection systems. Because different referral pathways were associated with different maltreatment profiles, reliance on a single sector or database is likely to underestimate the existing diversity and prevalence of child maltreatment experiences. Multidisciplinary institutions such as Childhood-Houses may therefore serve not only as assessment and support centers but also as important nodes for standardized documentation and data integration across sectors. Strengthening harmonized documentation procedures may improve both individual case management and the broader surveillance of child maltreatment within child protection systems.

Finally, both the routine documentation data and the survey findings suggest that psychological assessment and support may warrant further attention. Although the present study was not designed to evaluate service adequacy, the relatively limited use of psychological assessments and the recommendations provided by referrers indicate that psychological expertise may play an increasingly important role within multidisciplinary child protection settings. Locally, this is already addressed with higher equipped staff and ambulant psychotherapy offers.

Overall, the findings support the view that effective child protection depends on the interaction of multiple professional systems. Multidisciplinary structures such as Childhood-Houses may provide important opportunities to integrate these perspectives and facilitate coordinated responses to children affected by maltreatment.

## 5 Conclusions

The present study examined how children entered a multidisciplinary Childhood-House and whether referral pathways were associated with differences in identified maltreatment profiles and assessment procedures. The findings suggest that pathways into specialized child protection services are closely connected to the process of maltreatment recognition and documentation.

Children referred through different professional sectors presented with distinct patterns of identified maltreatment, indicating that healthcare providers, youth welfare services, and law enforcement agencies contribute complementary perspectives to child protection practice. Rather than representing parallel systems, these sectors appear to form interconnected components of a broader identification and referral network.

The findings further highlight the potential value of multidisciplinary service models that bring together children referred from otherwise separate professional contexts. By integrating information and expertise across sectors, Childhood-Houses may support a more comprehensive understanding of children's protection needs and facilitate coordinated assessment processes.

Future research should examine whether similar referral patterns can be observed across other Childhood-Houses nationally and Child Advocacy Center models globally and whether referral pathways influence subsequent support trajectories and outcomes. A better understanding of these mechanisms may contribute to earlier identification, more coordinated interventions, and improved support for children affected by ACE-related child maltreatment experiences.

## Data Availability

The data analyzed in this study is subject to the following licenses/restrictions: The datasets analysed in this study are not publicly available as they contain sensitive data relating to child protection. Requests to access these datasets should be directed to Silke Pawils, silke.pawils@med.uni-duesseldorf.de.
